# Induction-phase treatment costs for cryptococcal meningitis in high HIV-burden African countries: New opportunities with lower costs

**DOI:** 10.12688/wellcomeopenres.16776.1

**Published:** 2021-06-04

**Authors:** Bruce Larson, Amir Shroufi, Charles Muthoga, Rita Oladele, Radha Rajasingham, Alexander Jordan, Joseph N. Jarvis, Tom M. Chiller, Nelesh P. Govender

**Affiliations:** 1 Global Health, Boston University School of Public Health, Boston, MA, 02118, USA; 2 Health Economics and Epidemiology Research Office, Department of Internal Medicine, School of Clinical Medicine, Faculty of Health Sciences, University of the Witwatersrand, Johannesburg, South Africa; 3 CDC Foundation, Cape Town, South Africa; 4 Botswana Harvard AIDS Institute Partnership, Gaborone, Botswana; 5 College of Medicine, Univerity of Lagos, Lagos, Nigeria; 6 Division of Infectious Diseases & International Medicine, University of Minnesota, Minneapolis, Minnesota, USA; 7 Mycotic Diseases Branch, Centers for Disease Controls and Prevention, Atlanta, Georgia, USA; 8 Department of Clinical Research, Faculty of Infectious and Tropical Diseases, London School of Hygiene & Tropical Medicine, London, UK; 9 National Institute for Communicable Diseases, National Health Laboratory Service, Johannesburg, South Africa; 10 University of Witwatersrand, Johannesburg, South Africa

**Keywords:** HIV/AIDS, cryptococcal meningitis, induction phase, amphotericin B deoxycholate, flucytosine, liposomal amphotericin B, fluconazole, South Africa, Uganda, Botswana, Nigeria

## Abstract

**Introduction: **Access to and the cost of induction treatment for cryptococcal meningitis (CM) is rapidly changing. The newly-announced price for flucytosine ($0.75 per 500 mg pill) and possibly lower prices for liposomal amphotericin B (AmB-L) create opportunities to reduce CM treatment costs compared to the current standard treatment in low- and middle-income countries.

**Methods:** We developed an Excel-based cost model to estimate health system treatment costs for CM over a two-week induction phase for multiple treatment combinations, newly feasible with improved access to flucytosine and AmB-L. CM treatment costs include medications, laboratory tests and other hospital-based costs (bed-day costs and healthcare worker time). We report results from applying the model using country-specific information for South Africa, Uganda, Nigeria, and Botswana.

**Results:** A 14-day induction-phase of seven days of inpatient AmB-D with flucytosine, followed by seven days of high-dose fluconazole as an outpatient, will cost health systems less than a 14-day hospital stay with AmB-D and fluconazole. If daily AmB-L replaces AmB-D for those with baseline renal dysfunction, with a cost of $50 or less per 50 mg vial, incremental costs would still be less than the AmB-D with fluconazole regimen. Simple oral combinations (e.g., seven days of flucytosine with fluconazole as an inpatient) are practical when AmB-D is not available, and treatment costs would remain less than the current standard treatment.

**Conclusions**: Improved access to, and lower prices for flucytosine and AmB-L create opportunities for improving CM treatment regimens. An induction regimen of flucytosine and AmB-D for seven days is less costly than standard care in the settings studied here. As this regimen has also been shown to be more effective than current standard care, countries should prioritize scaling up flucytosine access. The cost of AmB-L based regimens is highly dependent on the price of AmB-L, which currently remains unclear.

## Introduction

Cryptococcal meningitis (CM) among people living with advanced HIV disease remains a leading cause of AIDS-related deaths globally. Meningitis deaths continue, in part, because of health system failures to diagnose and/or initiate patients on antiretroviral therapy quickly after HIV infection, and for patients who develop CM, failures to treat patients with efficacious induction-phase regimens including off-patent medications ^1– 9^. In short, the continued high incidence of CM cases and deaths are programmatic indicators of these failures ^2^.

This research note reports on the cost of treatment for CM patients during the initial two-week induction phase as access to key medications improves (and prices fall) and to complement new research evaluating effectiveness of alternative regimens containing combinations of amphotericin B deoxycholate (AmB-D), fluconazole, flucytosine, and liposomal amphotericin B (AmB-L) (see, e.g. 10, 11). In this analysis, treatment costs based on World Health Organization guidelines ^1^, include medications as well as laboratory tests and other hospital-based costs, which vary based on drug regimen. Using country-specific cost information, results are presented for Botswana, Nigeria, South Africa, Uganda.

Based on these country-specific analyses, if the announced price reduction for flucytosine is realized with adequate procurements, we conclude that AmB-D with flucytosine for seven hospital days (followed by fluconazole monotherapy in the second week as an outpatient) will cost providers (e.g., government health systems) substantially less than AmB-D plus oral fluconazole in hospital for 14 days. Costs for additional induction-phase regimens with AmB-L or simple oral combinations (flucytosine plus fluconazole) are also discussed.

## Methods

### Background

Prior to 2018, the WHO recommended two-weeks of hospital-based care with daily AmB-D infusions plus oral fluconazole as one of the preferred treatment options. This regimen became a standard treatment in many settings due to the lack of flucytosine regulatory approvals and limited access in most low- and middle-income countries (LMICs) ^1, 10, 12– 14^. The updated 2018 WHO guideline recommends AmB-D with flucytosine in place of fluconazole for week one and then high-dose fluconazole for week two ^1^. This regimen is more efficacious and, due to the shorter duration of AmB-D infusion, less toxic and allows for a shorter hospital stay. To date, flucytosine has been largely unavailable in LMICs despite being included in the WHO essential medicines list ^9, 10, 15^.

After years of advocacy ^8, 9, 16^, the lack of access to old and off-patent medications is beginning to change. Flucytosine is now available for $75 per 100 pack (500 mg pills) *ex works* although use in country remains very limited (mainly LMICs with a high HIV burden), while Gilead announced the company will seek to make AmB-L available for a substantially lower price as well ^17^.

### Model overview

We used a basic micro-costing approach, organized into an Excel-based model, to estimate per-protocol treatment costs from the health-system perspective (reported in 2019 $US) per CM patient and per 1,000 CM patients over a 14-day induction phase, where treatment costs include medications, laboratory tests and other hospital-based costs (bed-day/hotel cost and staff if not included in bed-day costs). Using the basic model, we completed four-country specific applications, which are available along with a User’s Guide at the OpenBU data repository ^18^. Any country-specific case study can also be used as a template for replication in other locations or with new assumptions.

The model first estimates cost for what has been a standard treatment across many LMICs; daily infusion of AmB-D for 14 days in hospital, if available, with high-dose oral fluconazole daily. Costs for a main alternative regimen, AmB-D with flucytosine for seven days (followed by fluconazole monotherapy in the second week), are then estimated along with additional regimens with AmB-L or simple oral combinations such as flucytosine plus fluconazole (in the absence of AmB-D or AmB-L) or fluconazole monotherapy, although effectiveness of this latter regimen is very low ^19^.

### Model structure and assumptions

The Excel model for each country contains the same five worksheets: table of contents; assumptions for all regimens; cost per patient for each regimen (seven total regimens are included); cost per 1,000 patients (which includes nine total regimens that consider alternative ways of addressing baseline renal dysfunction (RD) for patients as well as incident RD for a standard two regimen with AmB-D). All assumptions on resource quantities and unit costs for such resources are provided in assumptions for all regimens sheet and the cost by regimen per patient sheet. The model is adapted as needed for each country, for example based on medication price information (price per pill or per pack of pills, laboratory monitoring guidelines or practices, or information requiring inflation adjusting). 

Unit costs for all resources except flucytosine and AmB-L, all other medications, laboratory tests, therapeutic lumbar punctures, health worker time, hospital in-patient bed days, are based on country-specific sources (referenced within the Excel model application for each country-specific analysis). For flucytosine, we use the reported price ($75 per 100 pack of 500 mg pills) plus 25% to include additional shipping and handling costs ^16, 20, 21^. The cost of AmB-L remains uncertain at this time. In South Africa, for example, while the 2019 single exit price of AmB-L was $194 per 50 mg vial, a price of $16.25 per 50 mg vial has been reported ^22^ but currently remains unconfirmed by Gilead. For this analysis, we have used a price of $50 per vial (e.g., $40 *ex works* plus an additional 25% for shipping, handling, etc.). As procurement of these medication grows in the near future, better estimates will likely be available in the near future.

## Results

**Figure 1.**
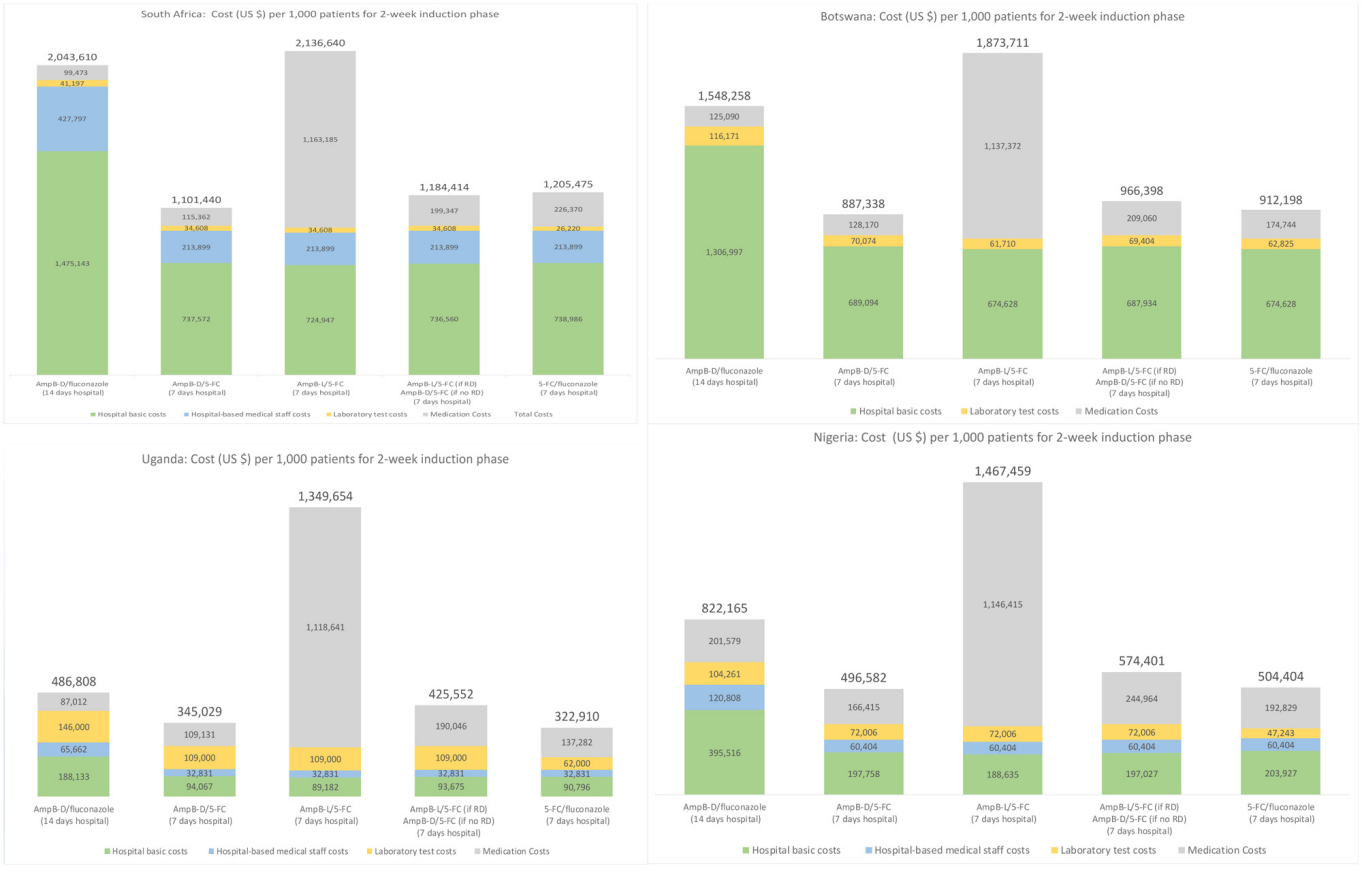
Cryptococcal meningitis treatment costs with alternative regimens ^*^. ^*^Total cost for the induction phase is provided at the top of each colored bar. The vertical axis (for costs) is not comparable (visually) across countries because the scale varies. For Botswana, hospital-based staff costs are included within the basic hospital costs.

Main results from these analyses are provided in Figure 1. For each country, five main treatment regimens are presented.

### AmB-D plus fluconazole (14 hospital days)

For each country, the first regimen reported in Figure 1 is a 14-day hospitalization with daily infusion of AmB-D with high-dose oral fluconazole daily. Given recommended daily dosages for this combination (50 kilogram adult; 1 mg/kg/day AmB-D; 1200 mg/day fluconazole) ^1^, medication costs per day are estimated at $7.11, $8.93, $6.22, and $14.40 for South Africa, Botswana, Uganda, and Nigeria, respectively. As summarized in Figure 1 (after dividing by 1,000), total costs per patient for this regimen are $2,043 (South Africa), $1,548 (Botswana), $822 (Nigeria) and $487 (Uganda). The basic hospital in-patient costs per day (excluding medications, and laboratory tests) in South Africa ($97) and Botswana ($88) are substantially higher than in Nigeria ($24) and Uganda ($11), which explains most of the differences between the higher- and lower-cost countries for this regimen. Treatment costs for this AmB-D/fluconazole regimen provide the reference point for discussing other regimens.

### AmB-D plus flucytosine (seven hospital days)

As included in the WHO 2018 guidelines, the preferred but previously unavailable combination is AmB-D/flucytosine for seven days followed by seven days of fluconazole. This regimen allows for seven hospital days among patients who do not need a more prolonged admission for other clinical reasons. With the newly-reduced daily cost for flucytosine at $9.38, this lower cost compares more favorable to the daily cost of fluconazole (e.g., the daily cost of 1200 mg fluconazole is estimated at $6.79, $0.43, $3.11, and $4.40 in South Africa, Uganda, Botswana, and Nigeria, respectively).

In all four country examples analyzed (see Figure 1), total costs with AmB-D/flucytosine (seven days) and then fluconazole monotherapy (seven days), with seven inpatient and seven outpatient days, are substantially less than with the AmB-D/fluconazole regimen. In each case, the additional daily medication costs for the first week (AmB-D with flucytosine instead of fluconazole) are offset by lower hospital costs and somewhat lower medication costs during week two (only fluconazole monotherapy).

### Replacing AmB-D with AmB-L

AmB-L is therapeutically equivalent and less toxic than AmB-D. Given the considerable morbidity associated with AmB-D infusion, benefits from improved access to AmB-L are clear. Assuming flucytosine is available, one option is to replace AmB-D with AmB-L and combine this with flucytosine during the seven hospital days. With dosing of 3 mg/kg/day and a patient weighing ≤50 kg, the daily cost for AmB-L is $150 per day. While significantly less than in the past, this daily cost would remain substantially higher than the daily cost of AmB-D during the induction phase ($0.31, $5.83, $5.78, and $10 for South Africa, Botswana, Uganda, and Nigeria, respectively).

From Figure 1, treatment costs with AmB-L/flucytosine compared to AmB-D/flucytosine during the first week of treatment (followed by fluconazole monotherapy in the second week for both regimens) increases significantly for all countries analyzed, while other costs largely remain the same. Additional research remains needed to consider how the possible benefits (ease of administration, side effects of medications, and treatment outcomes) of switching to standard doses of AmB-L for all patients might compare to the additional costs as well as the budgetary impact.

### Target AmB-L to patients with baseline renal dysfunction

One option to manage the costs of AmB-L, as included in the Southern African HIV Clinicians Society’s 2019 cryptococcal disease management guideline, is to target AmB-L/flucytosine to patients with known renal dysfunction at baseline, with AmB-D/flucytosine for the remainder, given that new AmB-D toxicities are uncommon in the first week of induction therapy ^23^. AmB-L is also a logical backup to manage AmB-D shortages or stock outs.

When the proportion of patients with renal dysfunction is ‘modest’, prioritizing these patients for AmB-L/flucytosine may be medically preferred and probably ‘affordable’ within the overall HIV care and treatment budget. For example, with 8% of CM patients with renal dysfunction (e.g., in South Africa estimated from 24), the total CM treatment cost per 1,000 patients would increase by about $83,000. With an estimated 21,000 new CM cases annually in South Africa ^2^, the annual additional cost of this approach would be $1.74 million annually, which is less than 0.12% of the $1.4 billion included in the national budget for 2019/2020 for the HIV and AIDS program budget ^25^. Given that new CM cases are, at least to some important degree, a consequence of health system failures, it seems logical for the program to internalize this cost of failures.

Note that the above discussion compares AmB-L and AmB-D when combined with flucytosine as part of a seven-day inpatient regimen. When compared to the standard 14-inpatient day regimen of AmB-D/fluconazole, however, treatment costs with AmB-L/flucytosine fall in Botswana and South Africa, increase substantially in Nigeria and modestly in Uganda.

### Oral regimens (flucytosine/fluconazole)

The WHO recommends an oral regimen of flucytosine/fluconazole (for 14 days) when AmB-D is not available. In Figure 1, costs for this regimen are included for seven inpatient days and seven outpatient days. Treatment costs with this oral regimen are similar to costs for AmB-D/flucytosine (lower costs from no daily infusions are offset by higher costs of the additional seven days of flucytosine). The cost of this flucytosine/fluconazole regimen would fall or increase depending on the number of days of inpatient care (e.g., only three or four days post-CM diagnosis to monitor intracranial pressure and other possible complications; or more if patients require ongoing management of raised intracranial pressure). The effectiveness of the alternative regimens, not just the costs, need to be addressed for a full comparison of the two regimens. In highly resource limited settings, however, the oral regimens make home-based care feasible at least for some subset of patients (i.e., those without severe CM at the time of treatment initiation, for example as measured by reduced level of consciousness).

## Conclusions

With flucytosine accessible at a price of $0.75 per 500 mg pill, an opportunity exists to reduce CM treatment costs over the initial two-week induction phase compared to standard care in LMICs (14 inpatient days with daily infusions of amphotericin B deoxycholate plus fluconazole). Although medication costs with flucytosine are higher than those of current standard treatment, cost reductions from fewer inpatient days (14 down to seven) more than offset the additional medication costs. Cost savings with flucytosine are substantial even in the examples presented in Figure 1 with lower hospital costs (Uganda and Nigeria).

If flucytosine is available, substituting AmB-L for AmB-D would involve substantial costs increases per patient if provided to all patients with CM. Nevertheless, the benefits of AmB-L (less toxicity and adverse reactions, easier administration, easier procurement and training to use one medication, etc.) warrant further analysis. One cost reducing strategy is to reserve use for patients presenting with renal dysfunction, who stand to gain the most from its use. In this case, AmB-L only to patients presenting with renal dysfunction, the incremental costs per 1,000 patients are modest in aggregate based on a cost of $50 per 50 mg vial. Clarity from Gilead on actual price(s) for AmB-L will allow for better cost estimates.

As new studies investigate new treatment strategies for CM cases, the costs for these new strategies can be easily estimated and compared using the costing model developed and used for this analysis. Such information on costs can then support discussions of budgetary impact and future economic evaluations of alternative treatment strategies.

## Data availability

### Underlying data

OpenBU: An Excel-based template for estimating induction-phase treatment costs for cryptococcal meningitis in high HIV-burden African countries. https://hdl.handle.net/2144/41876 ^18^.

This project contains the following underlying data:

• CM Induction Phase Treatment Costs -- Botswana May 17 2021.xlsx
• CM Induction Phase Treatment Costs -- Nigeria May 17 2021.xlsx
• CM Induction Phase Treatment Costs -- South Africa May 17 2021.xlsx
• CM Induction Phase Treatment Costs -- Uganda Dec May 17 2021.xlsx

### Extended data

OpenBU: An Excel-based template for estimating induction-phase treatment costs for cryptococcal meningitis in high HIV-burden African countries. https://hdl.handle.net/2144/41876 ^18^.

This project contains the following extended data:

• User_Guide_CM_treatment_costs May 17 2021.pdf

Data are available under a Creative Commons Attribution-NonCommercial 4.0 International license ( CC BY-NC 4.0).

## Disclaimer

The content of this manuscript is solely the responsibility of the authors and does not necessarily represent the official views of the CDC, NIH, NIHR, the Department of Health and Social Care, or other funding entities.
