# Supplementary material for: Induction-phase treatment costs for cryptococcal meningitis in high HIV-burden African countries: New opportunities with lower costs
Source: Wellcome Open Res. 2022 Jun 20;6:140. Originally published 2021 Jun 4. [Version 3] doi: 10.12688/wellcomeopenres.16776.3 (PMC9184925; doi:10.12688/wellcomeopenres.16776.3)
Supplement: Peer review history

# Peer review: Reviewer response for version 3

**DOI:** 10.21956/wellcomeopenres.19938.r51146

**Published:** 2022-07-25

**Authors:** Katharine Elizabeth Stott

**Affiliations:** 1 Antimicrobial Pharmacodynamics and Therapeutics, Department of Molecular and Clinical Pharmacology, Institute of Systems, Molecular and Integrative Biology, University of Liverpool, Liverpool, UK

No further comments - all comments/queries addressed by authors.

Is the work clearly and accurately presented and does it cite the current literature?

Partly

If applicable, is the statistical analysis and its interpretation appropriate?

Not applicable

Are all the source data underlying the results available to ensure full reproducibility?

Yes

Is the study design appropriate and is the work technically sound?

Yes

Are the conclusions drawn adequately supported by the results?

Partly

Are sufficient details of methods and analysis provided to allow replication by others?

Yes

Reviewer Expertise:

Antimicrobial pharmacology, PKPD, mycology

I confirm that I have read this submission and believe that I have an appropriate level of expertise to confirm that it is of an acceptable scientific standard.

# Peer review: Reviewer response for version 2

**DOI:** 10.21956/wellcomeopenres.19667.r50590

**Published:** 2022-06-09

**Authors:** Clinton J Pecenka

**Affiliations:** 1 Center for Vaccine Innovation and Access, PATH, Seattle, WA, USA

Summary

 This is a well written paper on the costs of cryptococcal meningitis treatment in African countries. New drug prices have been announced with may lower the costs of treatment compared to current front-line drugs. The analysis is methodologically sound and straightforward for the reader. Additional data could be included in the manuscript itself, but it also easily accessible in the Excel files. Given some uncertainty in drug prices, the primary value of this work may be subsequent analyses undertaken with the tools presented here.

 Introduction

 No comments

 Methods

 The following sentence could be simplified. As procurement of these medication grows in the near future, better estimates will likely be available in the near future.

 Results and discussion

 The authors could further note the value of reducing the need for inpatient services in some regimens. In many LMICs, hospital space is limited and reducing in facility resource requirements may allow additional patients, potentially with other illnesses, to be treated.

 Conclusion

 No comments

Is the work clearly and accurately presented and does it cite the current literature?

Yes

If applicable, is the statistical analysis and its interpretation appropriate?

Yes

Are all the source data underlying the results available to ensure full reproducibility?

Yes

Is the study design appropriate and is the work technically sound?

Yes

Are the conclusions drawn adequately supported by the results?

Yes

Are sufficient details of methods and analysis provided to allow replication by others?

Yes

Reviewer Expertise:

Global health economics, vaccine economics

I confirm that I have read this submission and believe that I have an appropriate level of expertise to confirm that it is of an acceptable scientific standard.

# Peer review: Reviewer response for version 2

**DOI:** 10.21956/wellcomeopenres.19667.r49536

**Published:** 2022-04-13

**Authors:** Angela Kairu

**Affiliations:** 1 Health Economics Research Unit, KEMRI-Wellcome Trust Research Programme Nairobi, Nairobi, Kenya

I have reviewed the revised article and Approve the revised version.

Is the work clearly and accurately presented and does it cite the current literature?

Partly

If applicable, is the statistical analysis and its interpretation appropriate?

Not applicable

Are all the source data underlying the results available to ensure full reproducibility?

Yes

Is the study design appropriate and is the work technically sound?

Yes

Are the conclusions drawn adequately supported by the results?

Yes

Are sufficient details of methods and analysis provided to allow replication by others?

Yes

Reviewer Expertise:

NA

I confirm that I have read this submission and believe that I have an appropriate level of expertise to confirm that it is of an acceptable scientific standard.

# Peer review: Reviewer response for version 1

**DOI:** 10.21956/wellcomeopenres.18501.r47861

**Published:** 2022-01-24

**Authors:** Katharine Elizabeth Stott

**Affiliations:** 1 Antimicrobial Pharmacodynamics and Therapeutics, Department of Molecular and Clinical Pharmacology, Institute of Systems, Molecular and Integrative Biology, University of Liverpool, Liverpool, UK

This paper addresses an important question for the long overdue implementation of flucytosine and amphotericin-B based induction treatment regimens for HIV-associated cryptococcal meningitis in LMICs – namely, the costs associated with those regimens. By comparing historically-recommended induction regimens to those currently recommended, the authors conclude that flucytosine and amphotericin-B-based regimens are affordable. This has positive implications for ongoing advocacy efforts to increase access to these essential medications.

 My comments mainly concern the clarity with which the analysis is presented and the rationale behind some of the analytical decisions. In addition, a number of assumptions are [necessarily] made and these should be highlighted more clearly.

Clarity

 The first two paragraphs of the methods section would be more appropriately included in the introduction section, since they provide background information.

 The authors state that they estimated costs for fluconazole monotherapy (second paragraph, ‘Model overview’). However, these estimates are not presented or stated in the paper, nor could I see them in the Excel downloads. I would recommend either including the data if they are available, or not stating that costs were estimated for fluconazole monotherapy, if these estimates are not available (in which case the authors might state why they decided against modelling those estimates - presumably because this regimen is not recommended).

Assumptions

 A major assumption made is that patients who are administered AmB-D plus 5FC for 7 days, followed by fluconazole for 7 days, are able to complete days 8 to 14 of their induction therapy as outpatients. Can the authors discuss what proportion of patients are able to be discharged from hospital after 7 days? Many patients may require ongoing inpatient care?

 As discussed by the authors, there is uncertainty surrounding the cost of AmB-L. In the absence of accurate cost information and because potential costs of AmB-L vary so widely, have the authors considered a highest and lowest potential cost calculation for the treatment strategies that contain AmB-L? i.e. offer a range of potential costs?

 I’m not sure I understand the third paragraph under ‘Target AmB-L to patients with baseline renal dysfunction’ in the results section (‘Note that the…’). I don't see that the statements made are reflected in figure 1? It appears to me that in all country case studies, the AmB-L/5FC option incurs the highest cost, with the option of AmB-L targeted to those with renal dysfunction incurring a lower cost than the reference regimen in all cases?

Additional comments

 Second sentence in Introduction section: deaths are also due to ART failure; data show that improved access to ART does not necessarily correlate with reduction in deaths from HIV-associated CM.

 Under Model structure and assumptions subheading: should ‘…incident RD for a standard two regimen…’ read, ‘…incident RD for a standard two ***week*** regimen…’?

Is the work clearly and accurately presented and does it cite the current literature?

Partly

If applicable, is the statistical analysis and its interpretation appropriate?

Not applicable

Are all the source data underlying the results available to ensure full reproducibility?

Yes

Is the study design appropriate and is the work technically sound?

Yes

Are the conclusions drawn adequately supported by the results?

Partly

Are sufficient details of methods and analysis provided to allow replication by others?

Yes

Reviewer Expertise:

Pharmacology, cryptococcal meningitis

I confirm that I have read this submission and believe that I have an appropriate level of expertise to confirm that it is of an acceptable scientific standard, however I have significant reservations, as outlined above.

# Response

**Published:** 2022-03-10

**Authors:** Bruce Larson

**Affiliations:** Boston University, USA

**Authors’ responses and revisions based on Reviewer Reports (on Version1)**

 In the following, we provide a point-by-point response to each comment and suggestion provided by Reviewer 2.  We have numbered each comment to ensure a complete response and to note information across comments and reviewers.

**Reviewer 2**

 24 Jan 2022 | for Version 1

 Katharine Elizabeth Stott, Antimicrobial Pharmacodynamics and Therapeutics, Department of Molecular and Clinical Pharmacology, Institute of Systems, Molecular and Integrative Biology, University of Liverpool, Liverpool, UK

 Approved With Reservations

 This paper addresses an important question for the long overdue implementation of flucytosine and amphotericin-B based induction treatment regimens for HIV-associated cryptococcal meningitis in LMICs – namely, the costs associated with those regimens. By comparing historically-recommended induction regimens to those currently recommended, the authors conclude that flucytosine and amphotericin-B-based regimens are affordable. This has positive implications for ongoing advocacy efforts to increase access to these essential medications.

 My comments mainly concern the clarity with which the analysis is presented and the rationale behind some of the analytical decisions. In addition, a number of assumptions are [necessarily] made and these should be highlighted more clearly.

 Clarity

 1. The first two paragraphs of the methods section would be more appropriately included in the introduction section, since they provide background information.

**Authors: Thank you for the recommendation.  We have revised and moved the paragraphs as recommended. Based on comments from the other Reviewer (Reviewer 1, comment 2), we deleted the original last paragraph of the introduction given that such information is also included in the conclusions section.**

 2. The authors state that they estimated costs for fluconazole monotherapy (second paragraph, ‘Model overview’). However, these estimates are not presented or stated in the paper, nor could I see them in the Excel downloads. I would recommend either including the data if they are available, or not stating that costs were estimated for fluconazole monotherapy, if these estimates are not available (in which case the authors might state why they decided against modelling those estimates - presumably because this regimen is not recommended).

**Authors.  Thank you for catching this omission. We originally included fluconazole monotherapy in our analysis, but then excluded given its poor effectiveness.  We have revised the Model Overview section to explain this point.**

 Assumptions

 3. A major assumption made is that patients who are administered AmB-D plus 5FC for 7 days, followed by fluconazole for 7 days, are able to complete days 8 to 14 of their induction therapy as outpatients. Can the authors discuss what proportion of patients are able to be discharged from hospital after 7 days? Many patients may require ongoing inpatient care?

**Authors: This is a very relevant point for real-world patient care.  Patients may need to remain in hospital even after their intravenous treatment is completed for continued management of raised intracranial pressure, for example. As noted in the introduction, the main focus of this Research Note is on treatment costs based on WHO guidelines. The 4 country-specific case studies (in the Excel files), however, can be used to address other questions and sensitivity analyses.  For example, as noted above for Reviewer 1, comment 3, future data from a clinical trial or monitoring patient care in routine settings might suggest that actual in-patient care typically extends to 10 inpatient days on average for the flucytosine plus fluconazole regimen. Such adjustments can be easily estimated using the models provided (e.g., cost of this regimen in South Africa for 7 in patient days is $1,101 and $1,484 for 10 days.  We have added this point into the main text of the paper (Section AmB-D plus flucytosine (seven hospital days)).**

 4. As discussed by the authors, there is uncertainty surrounding the cost of AmB-L. In the absence of accurate cost information and because potential costs of AmB-L vary so widely, have the authors considered a highest and lowest potential cost calculation for the treatment strategies that contain AmB-L? i.e. offer a range of potential costs?

**Authors: We have added a last paragraph into the AmB-L section to include the recommendation of the reviewer.  “While a substantially lower price ($16.25 per vial) has been reported by advocacy organizations (e.g.,** **https://www.gaffi.org/gilead-reduces-price-of-ambisome-liposomal-amphotericin-b-for-cryptococcal-meningitis-in-hiv-aids/** **), this price has not yet been confirmed by Gilead. Obviously, costs would fall substantially for this regimen with the lower price. For example, costs per day for AmB-L would fall from $150 to $60 per patient with this cost (a low-end estimate), but overall costs would remain substantially higher than with AmB-D.”**

 5. I’m not sure I understand the third paragraph under ‘Target AmB-L to patients with baseline renal dysfunction’ in the results section (‘Note that the…’). I don't see that the statements made are reflected in figure 1? It appears to me that in all country case studies, the AmB-L/5FC option incurs the highest cost, with the option of AmB-L targeted to those with renal dysfunction incurring a lower cost than the reference regimen in all cases?

**Authors: Thank you very much for highlighting the confusing paragraph.  We have revised the section to clarify the comparisons being made.  The AmB-L to patients with baseline renal dysfunction approach is the fourth option provided in Figure 1, which is mainly compared to the second option in Figure 1 AmB-D plus flucytosine for all patients. We then note that when compared to the 14-day regimen of AmB-D plus fluconazole (first regimen in Figure 1), costs for this fourth regimen are lower per 1,000 patients.**

 Additional comments

 6. Second sentence in Introduction section: deaths are also due to ART failure; data show that improved access to ART does not necessarily correlate with reduction in deaths from HIV-associated CM.

**Authors: We agree with the reviewer’s comment.  We have revised this sentence to include diagnose, initiate, retain, and achieve viral suppression…. We think the addition of “retain and achieve viral suppression” includes a broader set of reasons for ART failure.**

 7. Under Model structure and assumptions subheading: should ‘…incident RD for a standard two regimen…’ read, ‘…incident RD for a standard two week regimen…’?

**Authors: Yes.  Thank you.  We have revised.**

# Peer review: Reviewer response for version 1

**DOI:** 10.21956/wellcomeopenres.18501.r46070

**Published:** 2021-10-11

**Authors:** Angela Kairu

**Affiliations:** 1 Health Economics Research Unit, KEMRI-Wellcome Trust Research Programme Nairobi, Nairobi, Kenya

This paper succinctly describes different treatment regimens for cryptococcal meningitis in HIV patients and their associated costs. This paper highlights the use and costs of the different drug regimens against the context of resource constraints and patient contraindications of some of the medications.

 My main comments are in the structuring of the various sections in the paper, and description of study methods.

 The introduction gives a brief summary of the disease and outcomes. However, the 2nd and 3rd paragraph capture information that would better fit in the methods and conclusion sections respectively. Additionally, the introduction does not include any literature (past/current) on the drug regimens being costed. Perhaps the background provided in the methods sections would better fit in the introduction.

 The methods provide a good overview of the costing model and assumptions used (additional files). However, some details on costing methods are not reported as these ensure replicability of the work. A useful guide for this may be (Cunnama L, Garcia Baena I, Laurence Y, Sweeney S, Vassall A, Sinanovic E et al. Costing guidelines for tuberculosis interventions. Geneva: World Health Organization; 2019. Licence: CC BY-NC-SA 3.0 IGO ^1^)

 For the hospital based costs, which level of health facilities were sampled and costed? Or was this based on secondary data? This is not clear.

 In addition it may be beneficial to conduct a sensitivity analysis on the uncertain cost parameters given the cost variations across the countries. This will strengthen the analysis and the results of the work.

 The results and discussion sections are merged together which generally interpret the costs across the study countries. However, there are no comparisons to similar settings on the same drug regimens which would give a comprehensive understanding of the cost estimates and the variances. This ties to the limited literature of the drug regimens that is provided in this paper. Also, important to include is the limitations and strengths of this analysis.

Is the work clearly and accurately presented and does it cite the current literature?

Partly

If applicable, is the statistical analysis and its interpretation appropriate?

Not applicable

Are all the source data underlying the results available to ensure full reproducibility?

Yes

Is the study design appropriate and is the work technically sound?

Yes

Are the conclusions drawn adequately supported by the results?

Yes

Are sufficient details of methods and analysis provided to allow replication by others?

Yes

Reviewer Expertise:

economic evaluations of healthcare programmes

I confirm that I have read this submission and believe that I have an appropriate level of expertise to confirm that it is of an acceptable scientific standard, however I have significant reservations, as outlined above.

## References

[1] : Costing guidelines for tuberculosis interventions. *WHO* .2019;

# Response

**Published:** 2022-03-10

**Authors:** Bruce Larson

**Affiliations:** Boston University, USA

**Authors’ responses and revisions based on Reviewer Reports (on Version1)**

 In the following, we provide a point-by-point response to each comment and suggestion provided by Reviewer 1.  We have numbered each Reviewer comment to ensure a complete response and to note information across comments and reviewers.

**Reviewer 1:**

 11 Oct 2021 | for Version 1

 Angela Kairu, Health Economics Research Unit, KEMRI-Wellcome Trust Research Programme Nairobi, Nairobi, Kenya

**Approved With Reservations**

 1. This paper succinctly describes different treatment regimens for cryptococcal meningitis in HIV patients and their associated costs. This paper highlights the use and costs of the different drug regimens against the context of resource constraints and patient contraindications of some of the medications.

**Authors:  Thank you for your efforts and guidance on this manuscript.  In the following, we will respond to questions and explain how and where we have revised the manuscript based on the Reviewers comments (Dr. Kairu and Dr. Stott).**

 My main comments are in the structuring of the various sections in the paper, and description of study methods.

 2. The introduction gives a brief summary of the disease and outcomes. However, the 2nd and 3rd paragraph capture information that would better fit in the methods and conclusion sections respectively. Additionally, the introduction does not include any literature (past/current) on the drug regimens being costed. Perhaps the background provided in the methods sections would better fit in the introduction.

**Authors:  Thank you for these comments.  As recommended, we moved the “Methods Background” section to the introduction, which also provides additional literature on drug regimens. Also as suggested, we removed the last paragraph of the introduction from the introduction section.  In hindsight, the information in that paragraph is essentially already in the conclusions section, so we just deleted it to avoid duplication. We hope it is acceptable to leave the original paragraph two in the introduction (now the final paragraph).  We think it helps the reader to understand the main objective of the paper and what’s to come.**

 3. The methods provide a good overview of the costing model and assumptions used (additional files). However, some details on costing methods are not reported as these ensure replicability of the work. A useful guide for this may be (Cunnama L, Garcia Baena I, Laurence Y, Sweeney S, Vassall A, Sinanovic E et al. Costing guidelines for tuberculosis interventions. Geneva: World Health Organization; 2019. Licence: CC BY-NC-SA 3.0 IGO1)

**Authors:  As recommended, we added the reference to Cunnama et al. along with the Drummond book (also a standard reference for costing methods).  Regarding replication, all cost-related information, assumptions, and results for this manuscript come directly from the country-specific analyses (one Excel file per country) provided in the OpenBU repository referenced in this manuscript.**

**As importantly, any of these country-specific analyses can be used as a template for replication and adaptation in other contexts.  For example, in the future, data from a clinical trial or observational study might suggest that actual in-patient care in that study extended from 7 to 10 inpatient days on average for the flucytosine plus fluconazole regimen. Such adjustments can be easily incorporated into the analysis for a study-specific analysis.**

 4. For the hospital based costs, which level of health facilities were sampled and costed? Or was this based on secondary data? This is not clear.

**Authors:  Because this article is intended as a Research Note, we did not review all of the specific assumptions used in each of the country-specific analyses. As noted in the section “Model structure and assumptions”, such details can be found in the Excel file for each country.  For example, for South Africa, the hospital cost assumptions (see sheet “Assumptions for all regimens”) are in cells D63-D67 (and reference is in cell G63). In this base analysis presented in the paper, the hospital bed-day cost was 50% of the national average bed-day cost, which is considered a conservative assumption when comparing costs for different regimens.**

 5. In addition it may be beneficial to conduct a sensitivity analysis on the uncertain cost parameters given the cost variations across the countries. This will strengthen the analysis and the results of the work.

**Authors:  Because this article is intended as a Research Note, we did not include additional discussion based on sensitivity analyses.  However, as noted in the section “Model overview”, the Excel files for any country can be used as a template for additional analyses and for readers to explore the sensitivity of results to assumptions. For example, in the South Africa file, the base case hospital cost assumption is chosen as “50% of the national average” (cell c659 is 4).  The file contains four other hospital cost possibilities that could be used for sensitivity analyses (the same structure exists in all the Excel files).  We added additional information on conducting sensitivity analyses at the end of the methods section.**

 6. The results and discussion sections are merged together which generally interpret the costs across the study countries. However, there are no comparisons to similar settings on the same drug regimens which would give a comprehensive understanding of the cost estimates and the variances. This ties to the limited literature of the drug regimens that is provided in this paper. Also, important to include is the limitations and strengths of this analysis.

**Authors:  For each country, we consider AmB-D plus fluconazole with 14 in-patient days as reference point for discussing the results for the other regimens.  This regimen is the comparison for the same setting for the other regimens evaluated (and presented in Figure 1).  We have retitled the Results section to Results and Discussion to note the combination, which we think in this case makes sense. The focus here is on medications and combinations that are already recommended by the WHO (see reference 1 that provides substantial review of medications, etc.).**
